# Rapid assessment of peripheral visual crowding

**DOI:** 10.3389/fnins.2024.1332701

**Published:** 2024-03-25

**Authors:** Dilce Tanriverdi, Frans W. Cornelissen

**Affiliations:** Laboratory for Experimental Ophthalmology, University of Groningen, University Medical Center Groningen, Groningen, Netherlands

**Keywords:** visual crowding, peripheral vision, eye movements, psychophysical techniques, adaptive psychophysics, forced-choice paradigms, serial search paradigm

## Abstract

Visual crowding, the phenomenon in which the ability to distinguish objects is hindered in cluttered environments, has critical implications for various ophthalmic and neurological disorders. Traditional methods for assessing crowding involve time-consuming and attention-demanding psychophysical tasks, making routine examination challenging. This study sought to compare trial-based Alternative Forced-Choice (AFC) paradigms using either manual or eye movement responses and a continuous serial search paradigm employing eye movement responses to evaluate their efficiency in rapidly assessing peripheral crowding. In all paradigms, we manipulated the orientation of a central Gabor patch, which could be presented alone or surrounded by six Gabor patches. We measured participants’ target orientation discrimination thresholds using adaptive psychophysics to assess crowding magnitude. Depending on the paradigm, participants either made saccadic eye movements to the target location or responded manually by pressing a key or moving a mouse. We compared these paradigms in terms of crowding magnitude, assessment time, and paradigm demand. Our results indicate that employing eye movement-based paradigms for assessing peripheral visual crowding yields results faster compared to paradigms that necessitate manual responses. Furthermore, when considering similar levels of confidence in the threshold measurements, both a novel serial search paradigm and an eye movement-based 6AFC paradigm proved to be the most efficient in assessing crowding magnitude. Additionally, crowding estimates obtained through either the continuous serial search or the 6AFC paradigms were consistently higher than those obtained using the 2AFC paradigms. Lastly, participants did not report a clear difference between paradigms in terms of their perceived demand. In conclusion, both the continuous serial search and the 6AFC eye movement response paradigms enable a fast assessment of visual crowding. These approaches may potentially facilitate future routine crowding assessment. However, the usability of these paradigms in specific patient populations and specific purposes should be assessed.

## 1 Introduction

The diminished ability to recognize objects in the presence of clutter is referred to as “visual crowding” (Bouma, 1970). Human vision is fundamentally constrained by crowding, affecting our performance in various tasks, including object recognition, reading, driving, and visual search (Whitney and Levi, 2011). Moreover, peripheral crowding effects are reported to be more pronounced in people with certain visual or neurological deficits such as dyslexia, schizophrenia, nystagmus, macular degeneration, and glaucoma (Geiger and Lettvin, 1987; Chung and Bedell, 1995; Levi, 2008; Whitney and Levi, 2011; Kraehenmann et al., 2012; Gori and Facoetti, 2015; Wallace et al., 2017; Ogata et al., 2019; Pel et al., 2019; Tailor et al., 2021; Shamsi et al., 2022). Elevated crowding affects the daily life of these groups negatively. For instance, crowding significantly slows down the reading of individuals with dyslexia (Martelli et al., 2009; Gori and Facoetti, 2015; Pel et al., 2019). Likewise, glaucoma patients with elevated crowding may experience difficulties in recognizing faces (Stievenard et al., 2021). Thus, understanding peripheral crowding in connection to these developmental and visual impairments is crucial for comprehending the deficits and developing treatments (De Vries et al., 2018). Additionally, it aids researchers in gaining a better understanding of the mechanisms underlying crowding. However, due to the current reliance on time-consuming, attentionally demanding, and conventional psychophysical techniques, studying crowding in these patient groups presents an important challenge to clinicians and researchers alike (Levi, 2008; Pelli et al., 2016; Verghese et al., 2019).

If we would have faster and more intuitive crowding tests, routinely assessing peripheral crowding could become more feasible. For example, in ophthalmology, such an assessment could help to identify early indicators of vision problems in glaucoma (Ogata et al., 2019) or can be used to predict reading rate in dyslexia (Martelli et al., 2009). Currently, various approaches exist to assess and quantify crowding. However, current methods for assessing crowding often involve intricate instructions and manual inputs, making them impractical for regular clinical application. Typically, participants must maintain steady fixation during the trial (Chung et al., 2001; Pelli et al., 2004), making crowding measurements attentionally demanding. This is contrary to natural visual behavior, in which eye movements both affect and are affected by our perception (Hunt et al., 2019). Previous studies have used eye movements to assess functional vision in a faster and more intuitive manner (Grillini et al., 2018; Soans et al., 2021). Eye movements also have been used as a “built-in response method” to replace manual responses when measuring crowding (Huurneman et al., 2014; Yildirim et al., 2015; Pel et al., 2019) thus liberating participants from the need to use an external keypad or mouse to respond to stimuli. However, previous studies either employed conventional psychophysical methods that required a high number of repetitions (Yildirim et al., 2015) or omitted threshold measurements (Pel et al., 2019).

The most popular conventional method uses threshold measurements to locate a target in the presence of surrounding flanking items (Levi et al., 2002; Pelli et al., 2004). This method is useful for disentangling differences in the extent and magnitude of crowding. Adaptive psychophysical approaches have been employed to enhance efficiency in crowding assessments, aiming to reduce the number of trials required to reliably assess crowding (Pelli et al., 2016; Kalpadakis-Smith et al., 2022; Shamsi et al., 2022). These methods improve efficiency by adjusting the difficulty of tasks based on participants’ performance, thereby minimizing the number of trials needed. In contrast, conventional threshold measurements are time-consuming due to manual responses and the need for extensive repetitions (Levi, 2008; Pelli et al., 2016). Thus, integrating adaptive psychophysics into crowding assessments offers a proven avenue for enhancing efficiency and precision.

In our study, we introduce an innovative approach to crowding assessment that builds on such existing methodologies while introducing innovative elements. While trial-based forced-choice paradigms incorporating eye movement responses have been explored before (Yildirim et al., 2015), our study stands out by integrating them with adaptive psychophysics, which is a novel combination. Additionally, we introduce a new serial search paradigm that encourages continuous eye movement responses, distinguishing itself from conventional paradigms that often rely on static stimuli and discrete responses. This paradigm also incorporates adaptive psychophysics. Therefore, by integrating eye movements into both existing and novel paradigms, and combining this with adaptive psychophysics, our study expands upon existing research.

We compare these paradigms in terms of crowding magnitude, the time required to obtain reliable thresholds and the perceived demand of the paradigms by the participants. Crowding magnitude assesses the strength of the effect, reflecting how nearby stimuli impede target recognition. Crowding extent, although not explored here, delineates the spatial range of interference around the target stimulus (Levi, 2008). Additionally, we manipulate target-flanker similarity to explore its impact on crowding. This similarity, characterized by feature resemblance between the target and flankers, typically increases crowding magnitude (Andriessen and Bouma, 1976; Nazir, 1992; Levi et al., 1994; Chung et al., 2001; Levi et al., 2002). We anticipate consistent crowding magnitude across all paradigms. Moreover, we expect that paradigms leveraging eye movements will require less time and be preferred by participants. We also expect to see increased crowding in all paradigms as the target flanker similarity increases.

## 2 Materials and methods

### 2.1 Participants

Data was collected from 15 adult participants (mean age: 25 years, SD: 3.9 years, range: 19–32 years, 9 female). Pilot experiments carried out prior to the experimental phase, coupled with existing literature (Yildirim et al., 2015) addressing the effect of response type (manual vs. eye movement) on crowding magnitude, provided the rationale for the inclusion of a cohort comprising 15 participants. Seven of the participants received 15 euros worth of gift cards for their participation. All participants were naïve to the aim of the experiment. All participants declared to be healthy and had (corrected to) normal vision. If corrected to normal, participants wore their glasses or lenses while performing the experiment. Confidentiality and anonymity were ensured by saving the data using two-digit numbers that were randomly assigned to each participant. The study was approved by the ethical committee of the University Medical Center Groningen. All procedures were performed according to the declaration of Helsinki (World Medical Association, 2013).

### 2.2 Materials

#### 2.2.1 Equipment

In all experiments, stimuli were presented on a light-emitting diode (LED) backlight monitor (BenQ XL2540 with a refresh rate of 144 Hz and pixel resolution of 1920 × 1,080). The measured mean luminance of the screen was 52 cd/m^2^. MATLAB software was used on MacBook Pro (mid-2015) with macOS Monterey (12.3.1) operating system to execute the experiments. The visual stimuli were manipulated using functions coded on PsychToolbox, MATLAB (Brainard, 1997; Pelli et al., 2004). Eye position signals were recorded with a desk-mounted eye tracker (SR Research, Kanata, Ontario, Canada) at a sampling rate of 1,000 Hz. The EyeLink’s built-in nine-point calibration procedure was used to calibrate the eye tracker. The eye tracker was controlled and integrated into the experimental script using the Eyelink Toolbox (Cornelissen et al., 2002). Only one eye of the participant was recorded. The eye recorded was decided based on the eye that demonstrated optimal calibration during the calibration process for each participant. The display was viewed binocularly from a distance of 67 cm. A fixed head position was ensured by using a head and chin rest.

#### 2.2.2 Paradigms and stimuli

**Figure 1:**
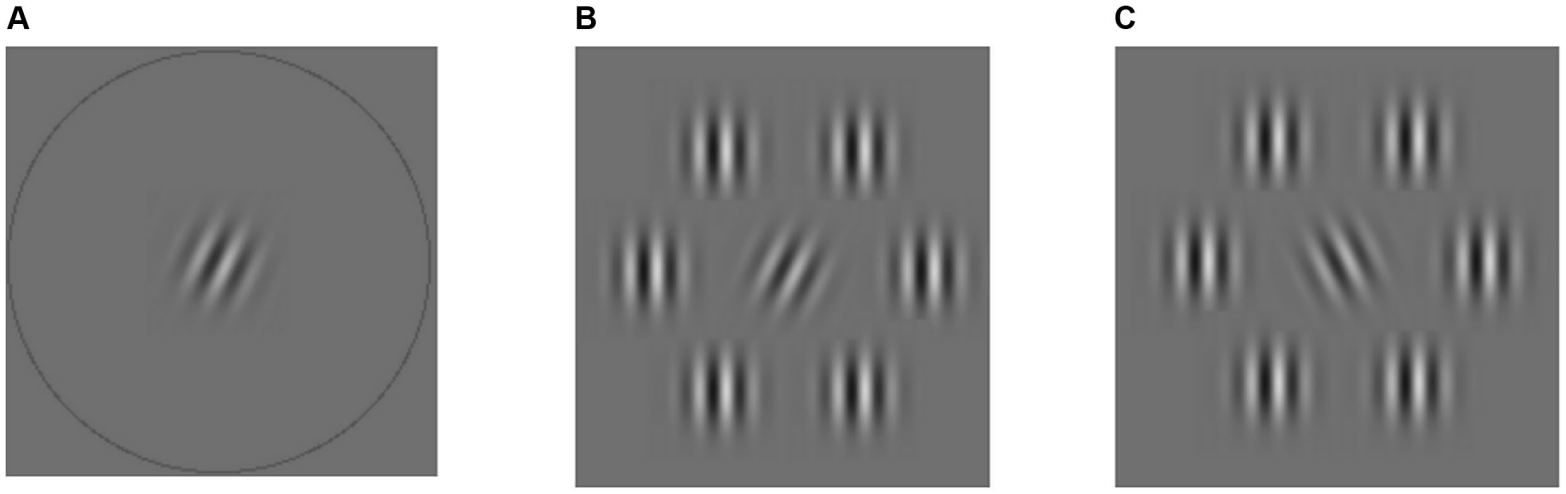
Stimulus elements used in different conditions. **(A)** A target in the isolated condition. **(B)** An example stimulus element with the target surrounded by flankers with the same spatial frequency as the target. **(C)** A non-target surrounded by flankers with the same spatial frequency. Both **(B,C)** are examples of elements used in the high target-flanker similarity condition. See Supplementary material for the low similarity condition example.

In all paradigms, a participant has to select a target among one or more non-targets. These stimulus elements consisted of a Gabor patch that could either be presented in isolation or surrounded by six Gabor patches that served as flankers. In case it was presented in isolation, this central element was surrounded by a circle (Figure 1A). The tilt angle of the central Gabors was manipulated. If it was tilted clockwise it served as the target (Figure 1B). If it was tilted anti-clockwise it served as the non-target (Figure 1C).

The spatial frequency of the flankers was manipulated to measure the effect of target-flanker similarity on crowding. We kept the target and non-target Gabors’ contrast lower than that of the flankers to enhance crowding effects in all paradigms (Chung et al., 2001; Felisberti et al., 2005) (see Table 1 for stimulus properties). The number of stimulus elements, as well as their position on the screen, varied depending on the paradigm. In all paradigms, the eccentricity of the stimulus elements was 6 degrees from the fixation point and the target-flanker distance was 1 degree center to center.

**Table 1:** Properties of different parts of a stimulus element.

Stimulus element parts	Size (diameter)	Contrast (Michelson-contrast)	Spatial frequency (cycles per degree)	Tilt-Angle (°)
Flankers	1°	75%	4 or 5°	90
Target	1°	50%	5°	90−(0–45)
Non-Target	1°	50%	5°	90 + (0–45)

### 2.3 Procedure

The study was conducted at the University Medical Center Groningen in a dimly lit room. Five different paradigms (two 2AFC paradigms with either manual or eye movement responses, two 6AFC paradigms with either manual or eye movement responses, and a continuous serial search paradigm) were presented in five separate blocks in random order. The relevant paradigm was explained to the participants at the beginning of each block and participants went through a training session to make sure they understood the current paradigm. The eye tracker was calibrated before each block. Participants were given a chance to rest their eyes without moving their heads after every 100 trials and could take a 10-min break after two or three blocks.

One block consisted of three conditions: isolated, high similarity flanked, and low similarity flanked. For each condition, an orientation discrimination threshold of the participant was determined using the QUEST at 75% correct performance (Pelli, 1987; King-Smith et al., 1994) with 100 valid responses. Conditions were interleaved and presented in random order. In total one block consisted of 300 valid trials.

After completing the experiment, participants filled out a questionnaire for each paradigm. To do this, participants were presented with a figure of each paradigm reminding them of the paradigm and the task associated with the paradigm. Participants were free to view these figures while filling out the questionnaires. The questionnaire consisted of five questions that were answered using a 1-to-5 Likert scale. The first question assessed the perceived difficulty of the paradigm, while the second inquired about participants’ level of tiredness upon completion of the paradigm. The third one aimed to gage the demands of the paradigm. The fourth one inquired about the perceived level of attention required during the paradigm, while the fifth and final question asked about the amount of effort required to complete the paradigm (see Supplementary material for the actual questionnaire). Participants were informed about the general objective of the study, which involved evaluating various methods for measuring peripheral crowding. However, they were not informed about the specific ideas behind each of the paradigms.

#### 2.3.1 2AFC and 6AFC paradigms

**Figure 2:**
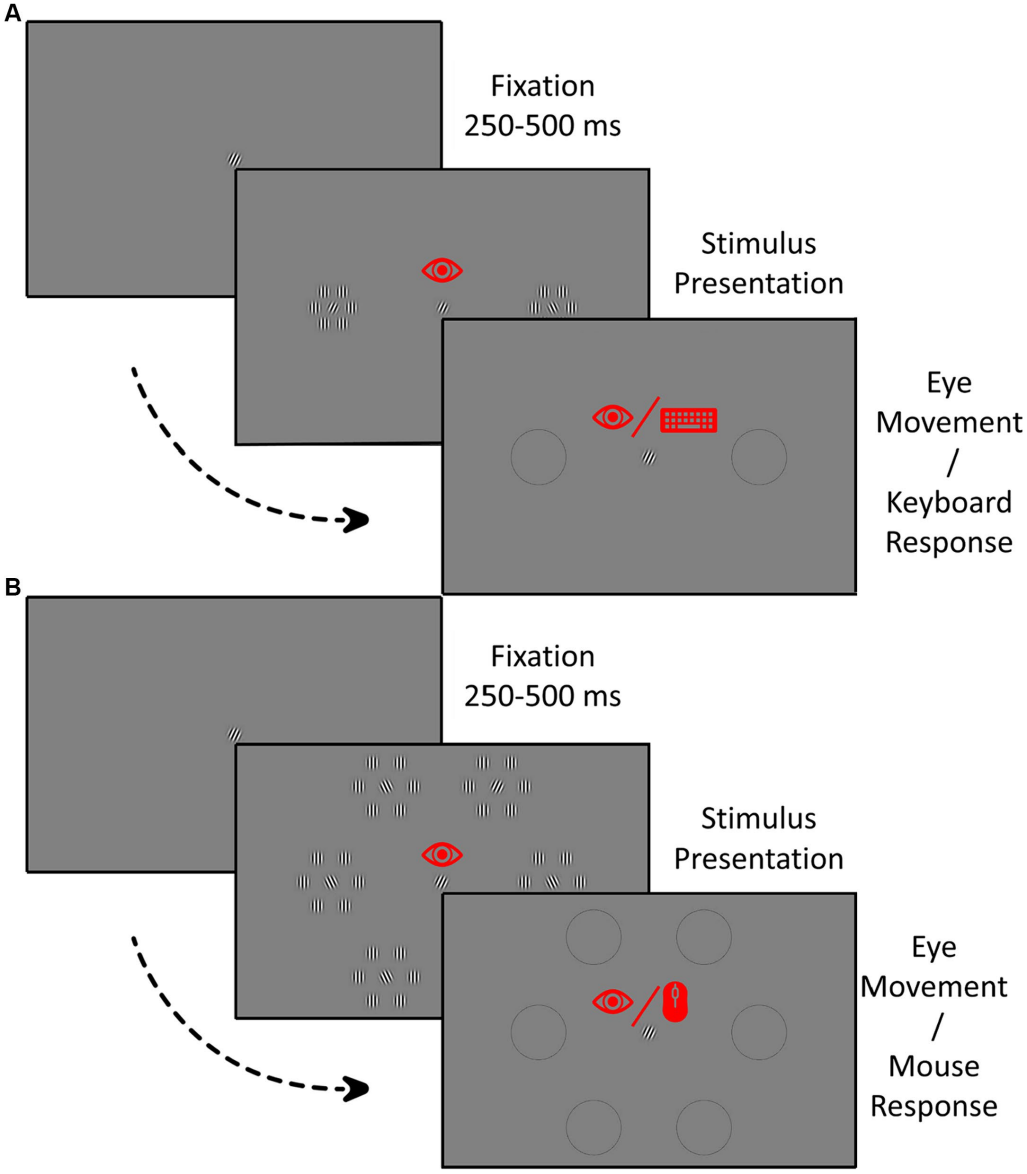
Procedure in the 2AFC paradigm **(A)** and the 6AFC paradigm **(B)** with both manual and eye movement responses. The eye icon indicates the requirement to fixate during the stimulus presentation panels and a saccadic response in the response panels. The keyboard icon in the response panel in **A** indicates a manual button press as a response while the mouse icon in **B** represents the need to point and click the mouse.

In the 2AFC paradigm, two stimulus elements were presented on the horizontal meridian at 6-degree eccentricity (see Figure 2). A reference Gabor was presented in the middle of the screen and remained visible throughout the experiment which served as the fixation spot. The target was placed randomly to the left or right of fixation. Upon establishing fixation using the eye tracker, and a subsequent random interval of 200–500 milliseconds, the stimuli were presented for 700 milliseconds. Participants were instructed to maintain fixation on the central Gabor during stimulus presentation and peripherally find the same Gabor in one of the two locations. If they broke fixation during the stimulus presentation, the target and the non-target were deleted from the screen. Following the stimulus presentation, thin black circles were displayed on the screen where the stimulus elements had been. In the manual response paradigms, participants responded by pressing either the left or the right-pointing arrow key on a keyboard. In the eye movement paradigms, they made a saccade to the location of the perceived target. Participants were required to respond only after the stimulus elements had disappeared for both manual and eye movement response paradigms. They did not have a time restriction to respond after the stimulus elements disappeared.

The 6AFC paradigm was similar to the 2AFC paradigm. However, in this paradigm, six stimulus elements were presented in a hexagonal shape, each at an eccentricity of 6 deg. In the 6AFC manual response paradigm participants moved the mouse to the perceived target location and confirmed their choice by pressing the mouse button. In the eye movement variant, the participant responded by making an eye movement to the perceived target location.

In both paradigms, the orientation discrimination threshold was estimated using QUEST. After every trial, we updated QUEST with the participant’s response and got a new tilt orientation recommendation for the upcoming trial. If the participant’s response was correct, the tilt orientation for the next trial would be smaller and if the response was false, the tilt orientation for the next trial would be bigger. For the 6AFC paradigm, the QUEST parameters were set to aim for 75% correct performance while assuming a guess rate of 0.17. For the 2AFC paradigm, the QUEST parameters were set to aim for 75% correct performance while assuming a guess rate of 0.5.

#### 2.3.2 Continuous serial search paradigm

**Figure 3:**
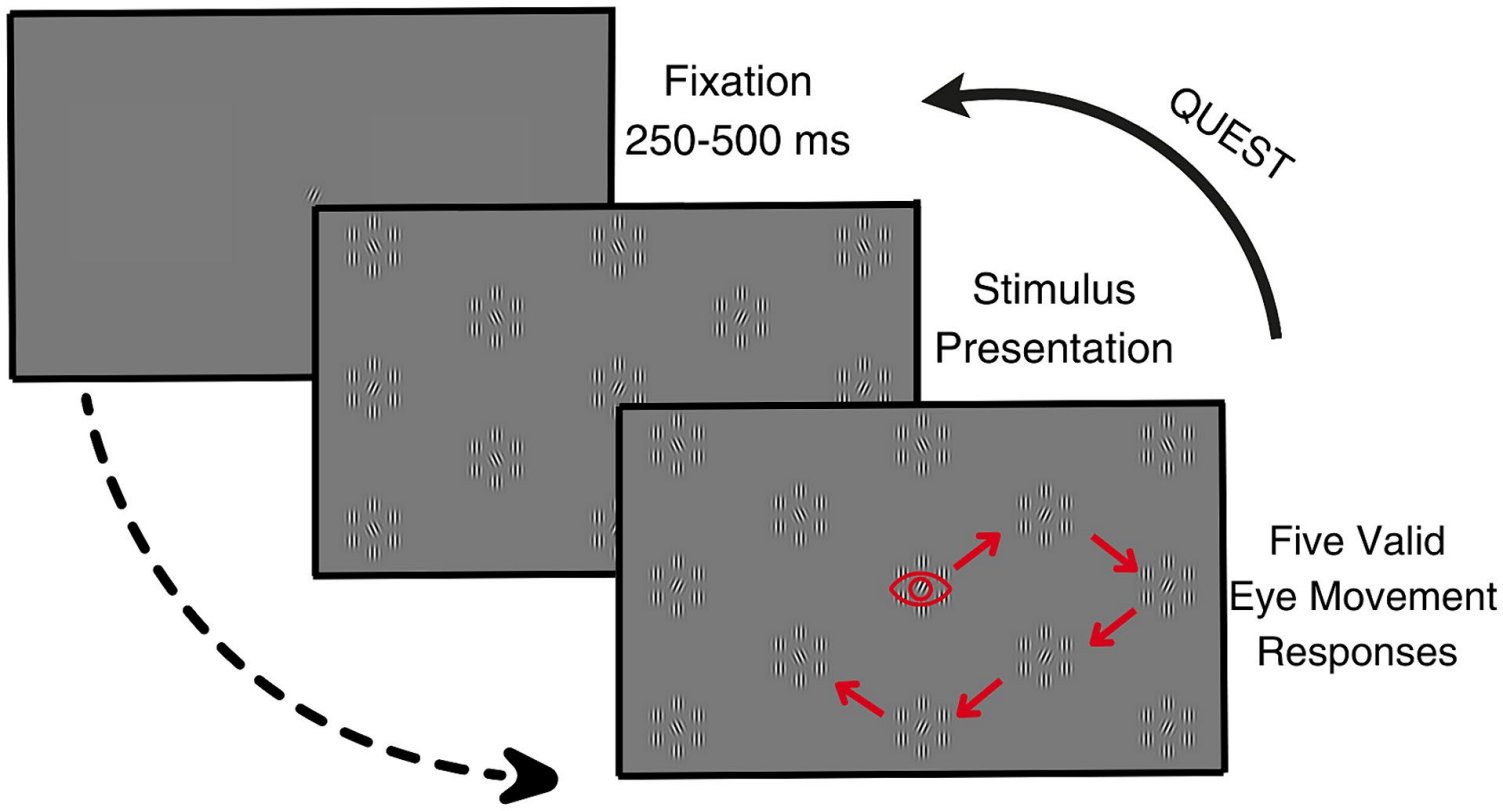
The procedure of the serial search paradigm. The eye icon represents the saccadic response. The arrows represent a hypothetical path a participant can take during the presentation of one target orientation. Note that the stimulus presentation shows only a part of the screen to increase the visibility of the image and not all of the stimulus elements.

In this paradigm, a grid of 28 stimulus elements was presented on the screen with equal center-to-center distances between elements (corresponding to a 6 deg. eccentricity of the central patches of elements). Half of the elements contained targets and the other half contained non-targets (see Figure 3). The stimulus elements containing targets were selected semi-randomly, such as to ensure that half the elements in a column contained a target and that the central element of the entire screen always had a target serving as a starting point. We ensured that at least one of the neighboring stimuli of this starting point included a target.

The experiment started with the presentation of a reference Gabor in the middle of the screen that illustrated the tilt of the target elements. Following 100 milliseconds of stable fixation on the reference, as established by on-line analysis of the eye tracking signals, and a subsequent random interval of 200–500 milliseconds, the stimulus screen was presented. Participants were instructed to continuously move their eyes to the elements on the screen where they perceived the presence of a target. An eye movement to a stimulus element counted as a valid response. If it was to a target, it was also counted as a correct response. If participants went back to a previously chosen stimulus element, the response was considered invalid and not counted. After five valid responses, the stimulus was refreshed: the reference was presented again, the locations of the target and non-target Gabors on the screen were randomized, and the orientations of the central Gabor patches were updated according to the value indicated by QUEST. Note that our approach resulted in QUEST receiving five responses in a row for the same orientation angle before being requested for a new orientation angle.

In this paradigm, the correct performance rate was set to 75% and the guess rate to assume by QUEST was set to 50%, which is the guess rate for the entire stimulus screen. We assumed this guess rate, even though it is not fixed for all locations on the screen. For example, at the center of the screen, participants can choose amongst 6 distinct elements. Yet, on the edges and in the corners of the screen, the number of possible elements to choose from is less. Nevertheless, we assumed a constant guess rate in order to avoid having to adjust the guess rate based on the participant’s momentary gaze location.

Importantly, once a stimulus element had been gazed at, it was removed from the “valid response” pool to prevent repetitive gazing at the same elements. Technically, if participants were able to remember exactly which elements they had visited, this approach would make the guess rate fluctuate slightly after each response. To minimize this effect, we updated the stimulus after five valid responses.

### 2.4 Eye movement analysis

Eyelink’s built-in 9-point calibration procedure was utilized to calibrate the eye movements before each block. Eye movements were recorded and analyzed in real-time during the experiment. In the forced choice paradigms, fixation was required to be within a 1-degree radius from the center of the screen for 100 milliseconds for the stimulus to appear on the screen. For the paradigms with eye movement response, at the location of each central Gabor patch, a rectangular area of interest (AOI) of 1-degree width and height was placed. When a participant’s eyes landed within an AOI, a response was recorded.

### 2.5 Statistical analysis and psychophysical analysis

After collecting the data, we refitted the orientation thresholds. This was to optimize the 2AFC and 6AFC QUEST structures, evaluating performance at equivalent points. The 6AFC paradigms were specifically refitted at 58.4% correct performance, instead of 75% used during data collection. Note that the presented tilt orientations were determined by QUEST assuming a 75% correct response level and could not be changed for the *post hoc* analysis. Altering the performance level led to negative threshold recommendations for some in the 6AFC paradigms due to our wide-10 to 60 QUEST range. To address this, we narrowed the range to 0–50 for a better fit with psychometric data. For consistency, we also refitted the 2AFC and serial search paradigms, while adjusting only the range parameter. For the 2AFC paradigms and the serial search paradigm, the guess rate was kept at 50% and the correct performance level at 75%. This also led us to be able to better compare the convergence rates for the threshold estimates in the different paradigms.

The data was analyzed using RStudio, MATLAB, and JASP. One of our aims was to determine whether the crowding estimates obtained using different paradigms were comparable to each other. This cannot be accomplished using conventional hypothesis testing since this only allows us to reject the null hypothesis (Wagenmakers et al., 2008). Therefore, we conducted Bayesian hypothesis testing using JASP. We report evidence in terms of the *BF10* (the Bayes Factor in favor of the alternative hypothesis) to indicate the likelihood of the data under the alternative hypothesis compared to the null hypothesis. We correct for multiple comparisons by multiplying the posterior odds (*PO*) with the value derived from the formula


$$\frac{1-0.5^{\frac{2}{m}}}{0.5^{\frac{2}{m}}}$$

where m represents the number of levels of a factor (Westfall et al., 1997). The Bayes Factor cannot be adjusted in this way; thus for the post-hoc comparisons we report the *PO* rather than the *BF10*. To report the main effects and interactions we report $BF_{\mathrm{incl}}$ (BF inclusion). $BF_{\mathrm{incl}}$ reflects the evidence for models with a particular effect, compared to all models without one. The presence of outliers and data normality were checked using Dixon’s Q test (Dean and Dixon, 1951) and the Shapiro–Wilk test (Shapiro and Wilk, 1965).

To assess differences between flanked and isolated thresholds we used Bayesian repeated-measures ANOVA. The within-observer factors considered were paradigm type (5 levels: 2AFC eye, 2AFC manual, 6AFC eye, 6AFC manual, and serial search) and condition (3 levels: isolated target, low target–flanker similarity, high target–flanker similarity).

We computed crowding magnitude by dividing the thresholds of the flanked conditions by the thresholds of the isolated condition. We then employed repeated-measures ANOVA with within-observer factors paradigm type and flanker mode (2 levels: low and high target–flanker similarity).

To compare the time to complete the various paradigms, we conducted a repeated-measures ANOVA with within-observer factor paradigm type to compare the time to complete the various paradigms. To compare questionnaire scores, we conducted Bayesian Wilcoxon signed-rank tests.

### 2.6 Re-estimating thresholds and paradigm duration based on confidence intervals

In our comparison of the paradigms, we also want to consider their efficiency. Amongst others, this will vary because the number of alternatives (targets vs. non-targets) to choose from differs between paradigms. Therefore, we determined, *post hoc*, the number of trials required to reach a reliable threshold. To do so, we determined *a posteriori* cut-off confidence interval (CI) by inspecting the mean CI plots for all paradigms. As a cut-off, we selected the CI at which the CIs had stabilized across all conditions and for all paradigms.

For each paradigm and participant, we determined the number of trials required to reach this CI for their threshold estimations. Based on these numbers, we re-estimated the thresholds by taking only the trials into account that were required to reach the CI cut-off. This involved providing the Quest with responses up to the point where the required number of trials was achieved. In addition, we recalculated the duration for each paradigm as follows:


$$\begin{array}{l} \mathrm{Recalculated}\;\mathrm{Duration}=\\ \mathrm{Original}\;\mathrm{Duration}\times \frac{\mathrm{Number}\;\mathrm{of}\;\mathrm{Trials}\;to\;\mathrm{Reach}\;CI}{\mathrm{Total}\;\mathrm{Number}\;\mathrm{of}\;\mathrm{Trials}} \end{array}$$

Because the cut-off was determined *a posteriori* and by eye, we also re-estimated the thresholds and durations with cut-offs at CIs that were 1 and 2 units higher. The results for these CIs are presented in the Supplementary Materials.

The same statistical procedures were used to analyze these re-estimated thresholds. Non-parametric Bayesian t-tests were performed to analyze the re-estimated durations due to the non-normal distribution of the data.

## 3 Results

A total of 15 participants completed the experiments and filled out the questionnaires. Three participants were identified as true outliers using Dixon’s D test (all three *p*’s < 0.05) for the crowding magnitude analysis. These outliers caused the threshold distributions of some of the conditions to be skewed. After removing these participants, the data was normally distributed. Moreover, as described in the stimulus section, to manipulate target-flanker similarity, we changed flanker spatial frequency from 5 to 4 cycles per degree. However, in hindsight, this manipulation was too subtle. Chung et al. (2001) reported a spatial frequency bandwidth for crowding of 2.7 octaves. Indeed, across all paradigms, Bayesian paired-sample t-tests indicated no differences in thresholds for the two target-flanker similarity conditions (all *POs* < 1). Therefore, we report on the thresholds obtained for the isolated and high target-flanker similarity conditions of 12 participants. In the duration and questionnaire analyses, we included all 15 participants and all three conditions. For completeness, in the supplementary material, we report on the thresholds for all 15 participants as well as those obtained in the low target-flanker similarity conditions. The results obtained are similar.

**Figure 4:**
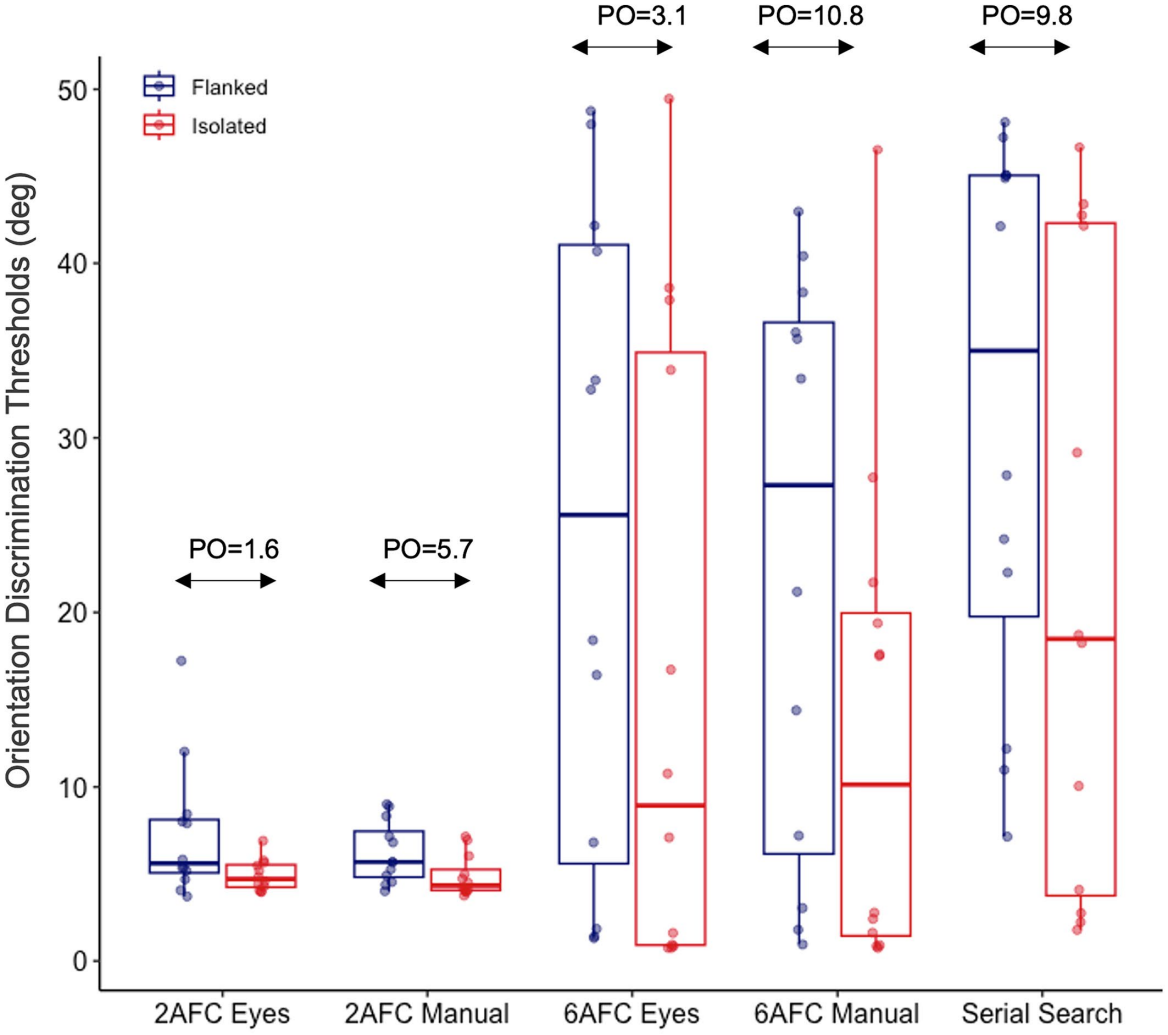
Isolated and flanked orientation discrimination thresholds obtained with all five paradigms. The figure shows flanked (high-similarity) orientation discrimination thresholds in blue and isolated orientation discrimination thresholds in red. “PO” represents the posterior odds of the flanked condition being different from the isolated condition for each paradigm. See Supplementary Figure S3 for the log scaled threshold values.

The results indicate a very strong influence of the paradigm type on thresholds ( $BF_{\mathrm{incl}}$ *=* 14385.75), a more modest influence of condition ( $BF_{\mathrm{incl}}$ *=* 1553.64) on thresholds, as well as an interaction between paradigm type and condition ( $BF_{\mathrm{incl}}$ *=* 41.44). Moreover, to determine the presence of crowding in each paradigm we compared the thresholds in the isolated to the flanked conditions using Bayesian paired sample t-tests. For all paradigms except the 2AFC paradigm with eye movement response, we found a difference between these conditions (see Figure 4).

Before conducting the repeated measures ANOVA for crowding magnitude we performed a log transformation on the crowding magnitude values, which served to normalize the skewed distribution of crowding magnitude values. After this transformation, the values were distributed normally. A comparison of the crowding magnitudes indicated that it was 33 times more likely than not that it differed across paradigms.

**Figure 5:**
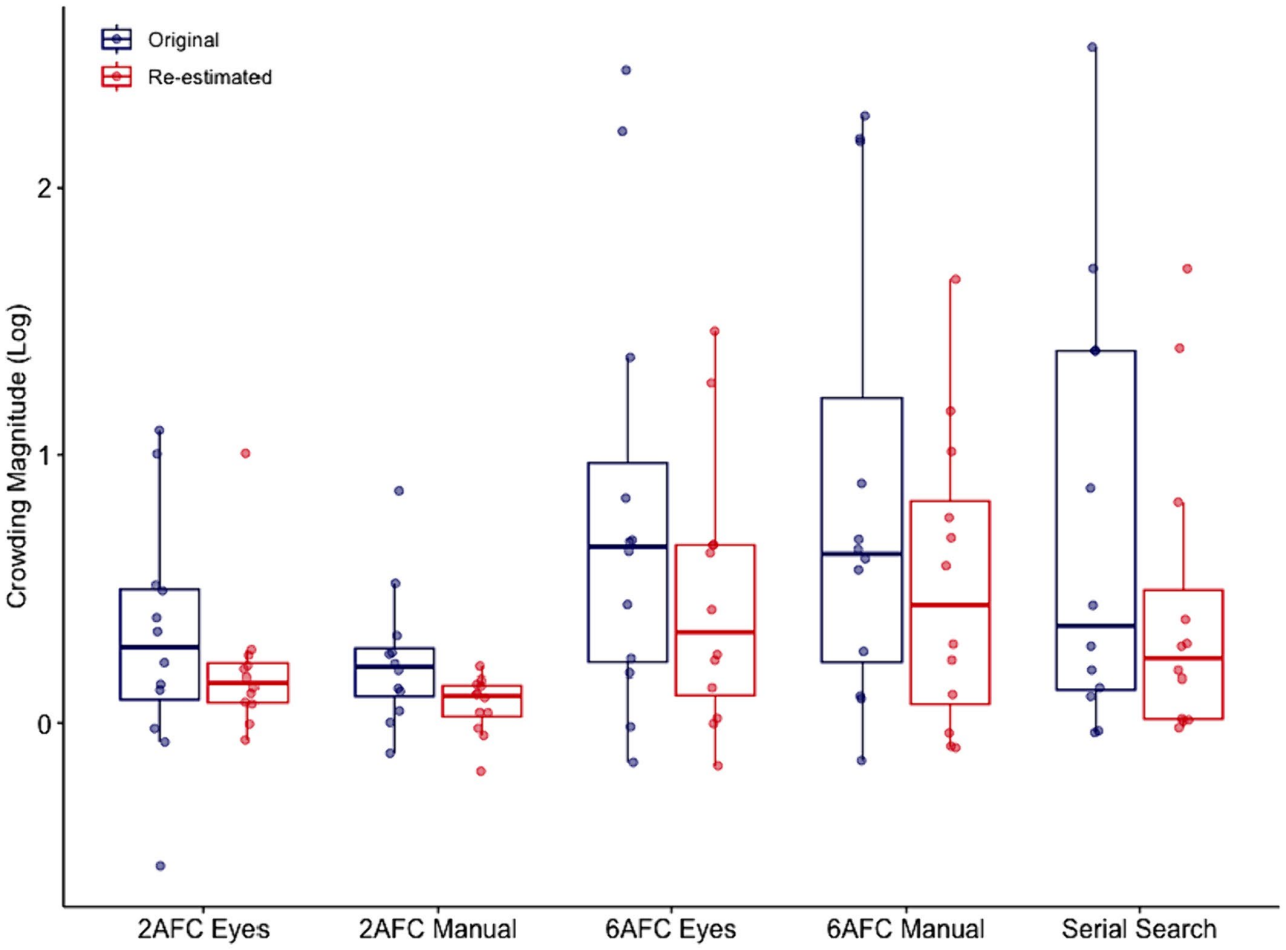
Comparison of the five paradigms in terms of crowding magnitude. Figure shows log transformed crowding magnitude values for the five different paradigms with 100 trials (blue symbols), and re-estimated crowding magnitude based on the number of trials needed for a reliable threshold (red symbols).

Post-hoc tests showed that crowding did not differ between the manual and eye movement response variants in either the 6AFC (*PO =* 0.09 *error* = 0.03) or the 2AFC paradigms (*PO =* 0.07 *error* = 0.02). Additionally, we observed similar levels of crowding in the continuous serial search and 6AFC paradigms, for both the eye movement (*PO =* 0.09 *error* = 0.03) and manual (*PO =* 0.08 *error* = 0.03) response variants of this paradigm. Moreover, we found anecdotal evidence for a difference in crowding between the continuous serial search and 2AFC paradigm with eye movement response (*PO =* 3.1, *error* < 0.01) but no evidence for a difference to the 2AFC paradigm with manual response (*PO =* 1.65, *error* < 0.01). Finally, crowding differed between the 2AFC and 6AFC paradigms, for both their eye movement (*PO =* 4.41, *error* < 0.01) and manual (*PO =* 6.47, *error* < 0.01) response variants (see Figure 5).

Both the crowding magnitude and orientation discrimination thresholds revealed individual variability within paradigms, specifically in the 6AFC and serial search paradigms. To see if this variability was consistent across paradigms we conducted correlation analyses between the paradigms for the isolated orientation discrimination thresholds and crowding magnitudes. Table 2 lists the correlation coefficient *r* and the associated *BF_10_* values describing the relationship between paradigms for either isolated thresholds and crowding magnitude. For the isolated thresholds, we found correlations between both 2AFC and 6AFC manual and eye movement paradigms. Moreover, for this measure, performance with the serial search paradigm was correlated with that in both 6AFC paradigms. Finally, we found a correlation between crowding magnitude in both the forced-choice eye movement paradigms and the serial search paradigm.

**Table 2:** Correlation (r) between paradigms for conditions with isolated thresholds, and the crowding magnitude.

Paradigms		Isolated thresholds		Crowding magnitude	
		*r*	*BF_10_*	*r*	*BF_10_*
2AFC Eye Movement	2AFC Manual	**00.58**	**2.01**	00.21	00.43
2AFC Eye Movement	6AFC Eye Movement	00.40	00.75	00.39	00.70
2AFC Eye Movement	6AFC Manual	00.08	00.36	00.49	1.13
2AFC Eye Movement	Serial Search	00.004	00.35	**00.76**	**12.39**
2AFC Manual	6AFC Eye Movement	00.48	1.07	−0.004	00.35
2AFC Manual	6AFC Manual	0.40	0.76	0.25	0.47
2AFC Manual	Serial Search	00.345	00.62	00.13	00.38
6AFC Eye Movement	6AFC Manual	**00.72**	**7.98**	00.34	00.59
6AFC Eye Movement	Serial Search	**00.62**	**2.71**	**00.77**	**16.16**
6AFC Manual	Serial Search	**00.70**	**6.48**	00.50	1.20

Values printed in bold indicate a *BF*_10_ that supports the correlation between the paradigms.

We collected the same number of trials in each paradigm and measured the time it took to complete it, thereby excluding any breaks. Our results revealed overwhelming evidence for an effect of paradigm type on experiment duration with the Bayes Factor going to infinity. See Table 3 for the *post hoc* comparisons.

**Table 3:** Posterior odds (PO) and error rates of post-hoc comparisons on assessment time between paradigms.

Paradigm comparison	PO	Error (%)
2AFC eye movement-serial search	12	<0.01
6AFC eye movement-serial search	444,498	<0.01
2AFC manual-serial search	1,205,000	<0.01
6AFC manual-serial search	1,073,000	<0.01
6AFC manual-6AFC eye movement	12	<0.01
2AFC Manual-2AFC Eye Movement	8,555	<0.01

**Figure 6:**
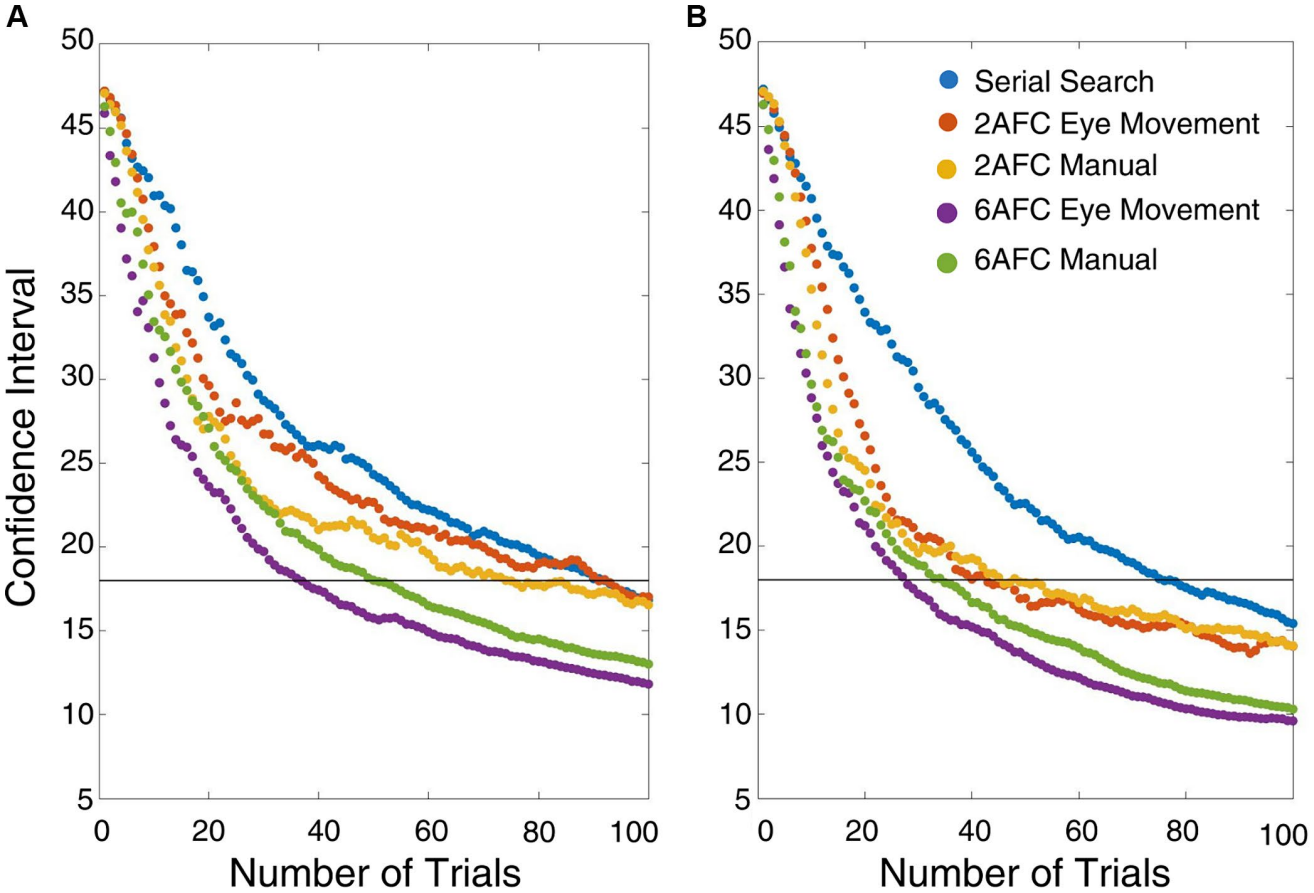
Change in confidence interval for the thresholds after each trial for each paradigm and condition. The black lines represent the selected CI cut-off (CI = 18). **(A)** High target-flanker similarity condition **(B)** isolated condition.

To take possible differences in the efficiency of the paradigms into account, we derived *a posteriori* cut-off confidence interval (CI) by inspecting the mean CI plots of all paradigms (Figure 6). Based on Figure 6, a cut-off CI value of 18 was chosen.

After adjusting the number of trials based on this CI, we verified whether this would have affected thresholds and crowding magnitude. We compared the original to recalculated thresholds using repeated measures ANOVA with the within-subject variables paradigm type, condition, and threshold calculation type (original vs. re-estimated). See Supplementary Figure S2 for the recalculated thresholds. Another repeated measures ANOVA with the within-subject variables paradigm type, condition, and threshold calculation type was run to see if there was a difference between the crowding magnitudes calculated using the original and re-estimated thresholds. Both ANOVAs revealed that re-estimated values were not different from the original values. See Table 4 for the $BF_{\mathrm{incl}}$ values.

**Table 4:** Bayes factors of main and interaction effects of Bayesian ANOVA on thresholds and crowding magnitude for comparison of re-estimated and original values.

**Effects**	Threshold $BF_{\mathrm{incl}}$	Crowding magnitude $BF_{\mathrm{incl}}$
Threshold calculation	0.94	2.92
Paradigm * Threshold calculation	2.55	0.77
Condition * Threshold calculation	0.28	0.41
Paradigm * Condition * Threshold calculation	0.59	0.002

**Figure 7:**
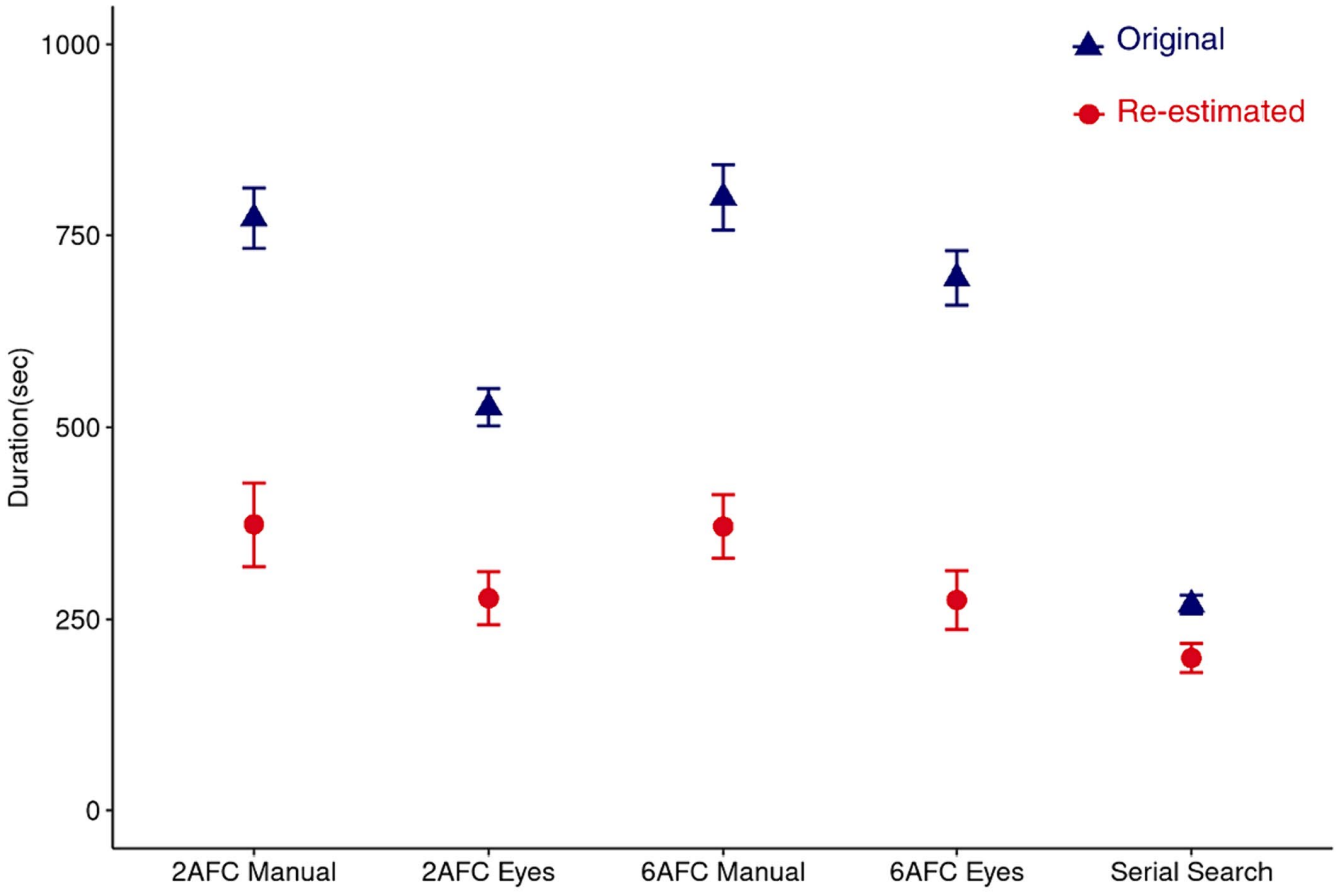
The amount of time required per paradigm to determine the thresholds with a fixed number of 100 trials (blue symbols) and after re-estimating the number of trials required to reach a reliable threshold measurement (red symbols).

Finally, we compared the adjusted durations using a Bayesian Wilcoxon signed-rank test due to the data not being normally distributed. Results revealed that based on the adjusted durations, participants took less time to complete the serial search paradigm compared to the 2AFC (*PO =* 28.13) and the 6AFC (*PO =* 9.54) paradigms with manual responses. However, it took a comparable amount of time for participants to complete the serial search and the 2AFC (*PO =* 1.20) and 6AFC (*PO =* 0.57) paradigms with eye movement responses. The difference in time taken by the manual and eye movement response paradigms persisted for the 2AFC (*PO =* 2.88) paradigms but disappeared for the 6AFC paradigms (*PO =* 0.47), see Figure 7.

**Figure 8:**
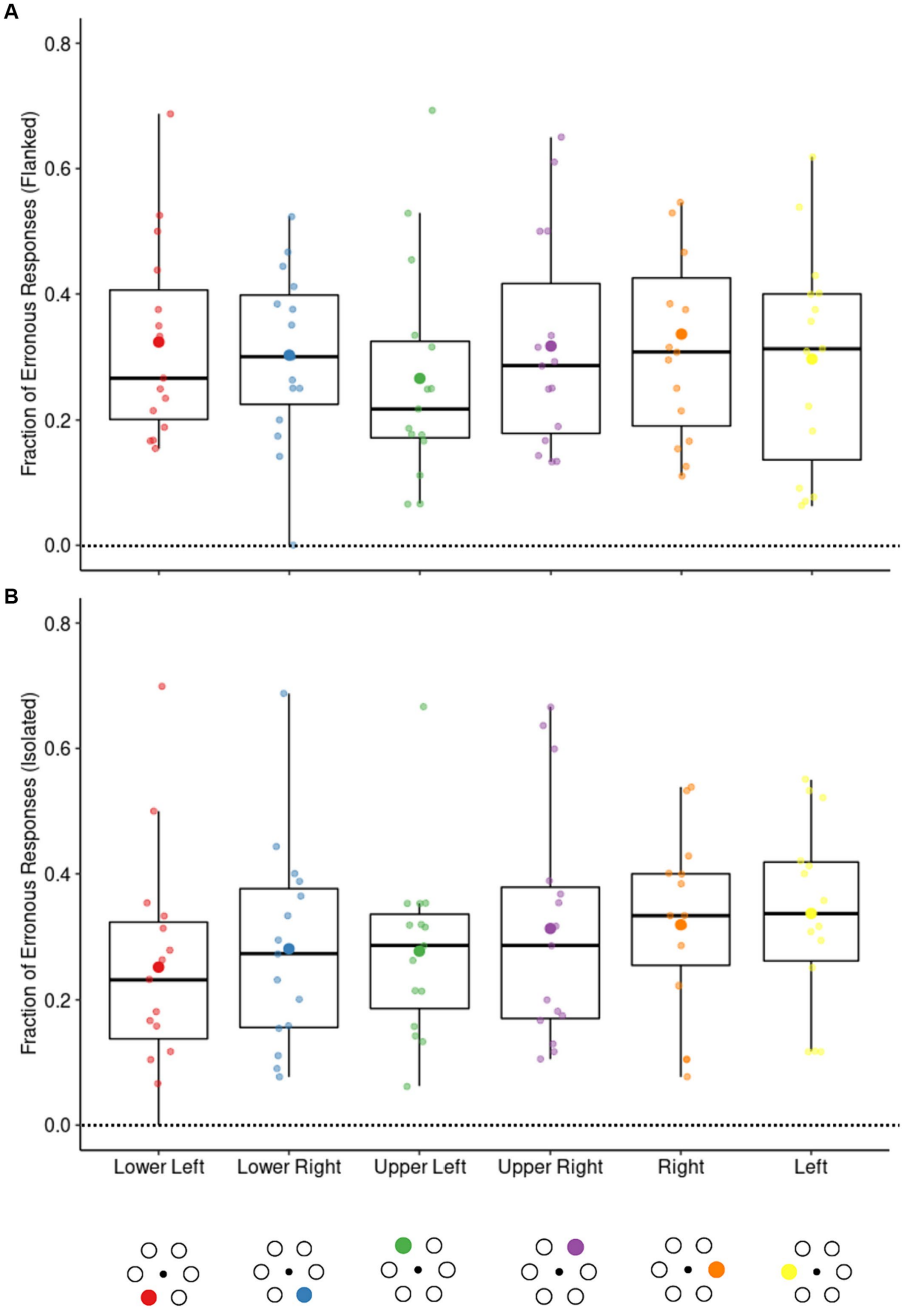
The fraction of errors in the flanked (high-similarity) and isolated conditions at each stimulus element location in the 6AFC eye movement paradigm. The filled circles below the figure represent the locations. The opaque dots on the box plots represent the mean while the boxplots show the median and IQR. One-way repeated measures ANOVA with location as a factor revealed no significant differences between locations for both the flanked **(A)** and the isolated conditions **(B)**.

The observed difference in crowding magnitude between the 2AFC paradigm on the one hand, and the 6AFC and serial search paradigms on the other, raised questions about the underlying factors shaping this difference. One possible explanation is that the crowding magnitude is affected by the position of the stimulus elements. The stimulus elements in the 2AFC paradigm were always horizontally oriented. In contrast, the stimulus elements in the 6AFC and serial search paradigms formed a hexagonal shape, resulting in stimulus elements placed both horizontally and diagonally concerning the fixation point. To test whether this may have affected our results we calculated the fraction of erroneous responses at each location in the 6AFC paradigm with eye movement responses for both flanked and isolated conditions. The results are shown in Figure 8. We found no evidence for a difference in erroneous responses based on stimulus location (all *POs* < 1).

**Figure 9:**
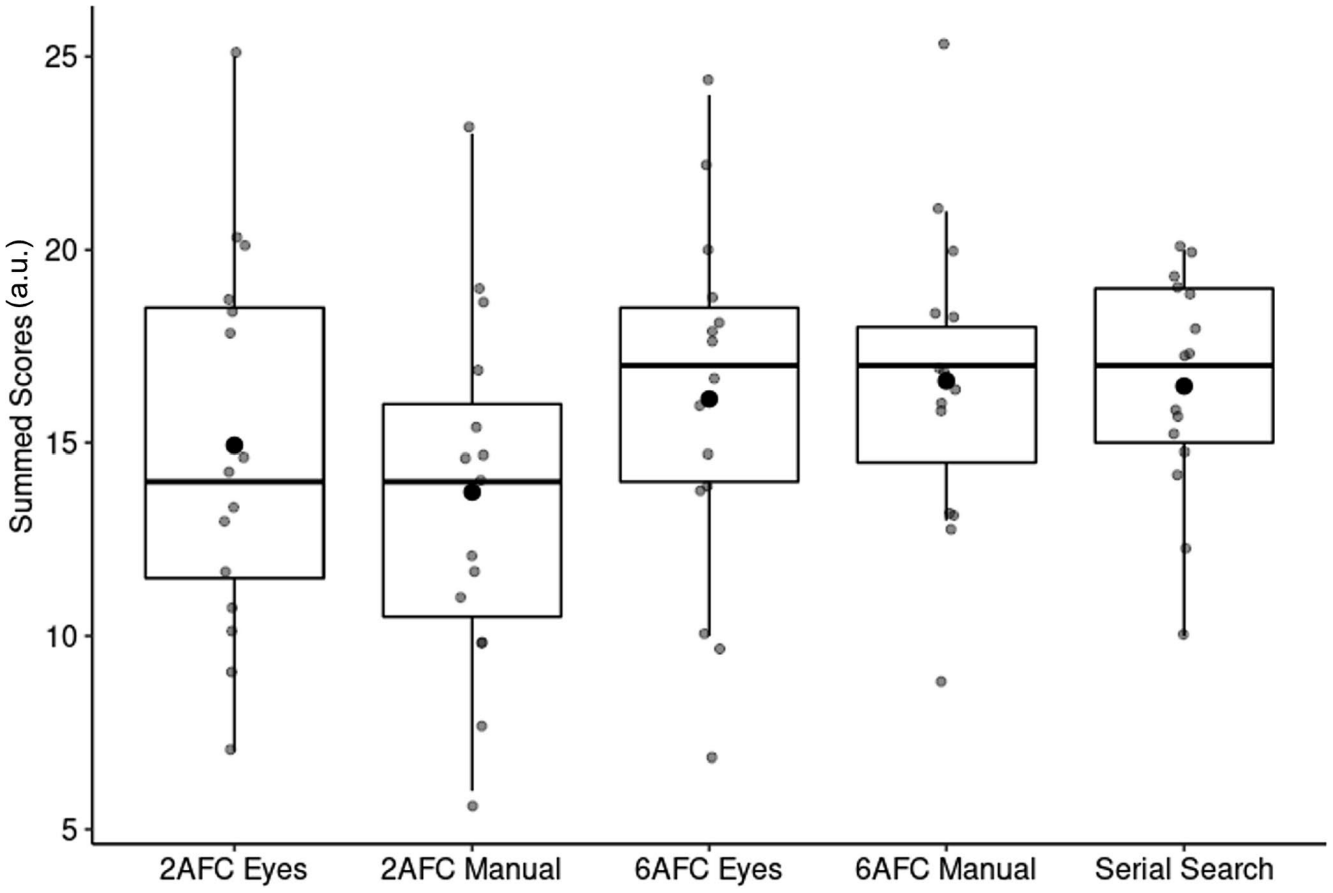
Demand (summed questionnaire scores) of each paradigm. The opaque dots on the box plots represent the mean while the boxplots show the median and IQR.

Finally, besides objective differences between the paradigms, we were also interested in any possible subjective ones as assessed by means of the questionnaire. We found no difference between paradigms (all *POs* < 1) in terms of the summed questionnaire scores (see Figure 9).

## 4 Discussion

In this study, we compared various paradigms for assessing peripheral visual crowding. Our main finding is that eye movement-based paradigms yield results faster compared to paradigms that necessitate manual responses. Furthermore, when considering similar levels of confidence in the measurements, both a novel serial search paradigm and an eye movement-based 6-Alternative Forced Choice (AFC) paradigm were most efficient for assessing crowding magnitude. Additionally, we found that the crowding estimates obtained through either of these two paradigms were consistently higher than those obtained using the 2AFC paradigms. Lastly, participants did not experience paradigms to differ in their demand. Below, we discuss these findings in more detail.

### 4.1 Eye-movement-based 6AFC and continuous serial search paradigms are most efficient in gaging crowding magnitude

The novel continuous serial search paradigm and the eye movement-based 6AFC paradigm emerged as the most rapid in estimating crowding magnitude. This was most noteworthy when we compared the paradigms while taking the confidence in the threshold estimates into account. Our results showed that these two paradigms exhibited the quickest assessment of crowding, while yielding similarly reliable threshold estimates and comparable magnitudes of crowding. Of these, the 6AFC paradigm requires fewest trials to reach a reliable threshold, owing to its lower chance level.

Thus, the use of eye movements as a response measure contributes to the increased efficiency of these paradigms. Unexpectedly, the 2AFC paradigms yielded lower crowding magnitudes compared to our 6AFC paradigms and the serial search paradigm. While we have no definitive explanation for this difference, we will discuss this finding in terms of an increased variability due to a larger number of possible target locations and a subsequent difference in the attentional demand and deployment of various paradigms.

Previous studies (Levi et al., 1994; Chung et al., 2001; Petrov and Meleshkevich, 2011; Greenwood et al., 2017; Kurzawski et al., 2023) suggest that critical spacing tends to be larger in the upper than in the lower visual field and stronger along the horizontal compared to the vertical meridian at the same eccentricity. Notably, in our 2AFC paradigm, the stimulus elements were arranged along the horizontal meridian in the 6AFC and serial search paradigms the stimulus elements were arranged in a hexagonal shape and placed along the horizontal meridian and diagonally in relation to the fixation point (where it has been shown that variations are less pronounced Abrams et al., 2012). An additional location analysis revealed that the errors made in the 6-AFC eye movement paradigm were unaffected by the specific location of the target stimulus.

An alternative explanation for the differences in crowding magnitudes between paradigms could be a difference in their spatial attentional demand. Previous research has indicated that attentional cues can influence crowding (Grubb, 2013; Kewan-Khalayly and Yashar, 2022; Kewan-Khalayly et al., 2022). It is arguably easier and likely more effective to attend to the two possible target locations of the 2AFC paradigms compared to the multiple locations in the other paradigms. This implies that participants could not follow the same covert attentional strategy in all paradigms. The cueing of location has been found to benefit both isolated and flanked performance (Scolari et al., 2007). Therefore, the different attentional demands may have increased the thresholds and the variability observed among individuals in the 6AFC and serial search paradigms, compared to the 2AFC paradigms.

### 4.2 Differential variability between paradigms and participants

Our data revealed increased variability between participants in 6AFC and serial search paradigms compared to the 2AFC paradigms. Large individual differences in crowding have been previously reported (Petrov and Meleshkevich, 2011; Greenwood et al., 2017) and are stable over time (Greenwood et al., 2017). To test if the variability amongst participants in our study was consistent over paradigms, we performed correlation analyses on the thresholds and crowding magnitudes. Our results revealed strong correlations between the two 2AFC paradigms, and between the 6AFC and serial search paradigms, on the isolated thresholds. Additionally, we found strong correlations between the 2-and 6AFC eye movement and the serial search paradigms for crowding magnitude. The latter correlations, along with comparable crowding magnitude levels observed in these paradigms that were substantially higher than those in the 2AFC paradigms, may support a role for attention in determining crowding magnitude. The correlation in performance on the 2-and 6AFC eye movement paradigms and the serial search paradigm confirms stable individual variability in crowding magnitude across these paradigms. Nonetheless, crowding magnitude becomes larger with an increase in the number of locations. The additional number of locations introduced in these paradigms may also have increased the variability between participants (Abrams et al., 2012) due to differences in the spatial attentional abilities of the participants. This may explain the absence of a correlation we observed in the isolated thresholds obtained with the 2AFC paradigms and the other paradigms. Note that a putative increased attentional demand in a paradigm need not to be a negative feature, as long as it amplifies relevant differences between participants. The correlation in crowding magnitude between the 2AFC and the serial search paradigm may suggest this to be the case. Irrespective, fully grasping this putative role of attention in crowding requires further experimentation.

### 4.3 Responding manually or by eye results in similar thresholds

Estimating crowding magnitude with either the 2AFC or the 6AFC paradigms yielded no discernible difference in thresholds obtained using manual and saccadic eye movement responses. This corroborates the findings of Yildirim et al. (2015), who reported similar results in 2AFC manual and eye-movement paradigms. Our study extends this observation to 6AFC paradigms, demonstrating the utility of eye movements as a valid measure for crowding assessment irrespective of the number of choices in a forced-choice paradigm. This result, coupled with the speed advantage of eye movement-based paradigms over manual response paradigms aligns with the notion of harnessing eye-movement behavior for efficient testing as has been suggested previously (Murray et al., 2009; Grillini et al., 2018; Pel et al., 2019; Soans et al., 2021). The reduction in assessment time for eye movement-based methods is relevant for both clinical and research settings, as quicker evaluations can enhance the feasibility of routine testing.

### 4.4 Participants do not experience differences in paradigm demand

Despite the demonstrated differences in efficiency, the participants themselves did not experience a difference in demand between the various paradigms. Plausible explanations for the absence of a clear participant preference could be our utilization of QUEST and the fixed number of trials used in each paradigm. As QUEST progresses and gains confidence in the threshold estimations, it presents values that are near the participant’s threshold. Consequently, all paradigms will eventually become equally challenging. Moreover, in our study, participants did not profit from the enhanced efficiency of the 6AFC paradigms, as they performed the same number of trials in all paradigms. The efficiency differences were established only in the post-hoc analyses. Additionally, our participant pool was relatively young and had a good visual and general health. In older participants and patients, differences in subjective experience with the various paradigms may be more pronounced, e.g., because of different attentional capacities.

### 4.5 Limitations and future directions

Our study provides insights into the efficiency of different paradigms for assessing peripheral visual crowding. The rapidity of the serial search paradigm and the eye movement-based 6AFC paradigm, combined with the comparable threshold estimates, implies a potential utility for streamlining routine crowding assessments. However, the increased variability in the 6AFC paradigms and serial search paradigms might be a pitfall when one tries to utilize these paradigms in clinical contexts. Our participant pool consisted mainly of young, healthy adults with normal visual function. Future studies should aim to replicate our findings in more diverse populations, including individuals with visual impairments, neurological disorders, and different age groups. Different patient groups have visual and cognitive differences that might differentially affect their performance in the various paradigms that are not present in our healthy and young participant sample. Such additional factors might increase the variability between individuals in these paradigms.

Thus, while eye movement-based paradigms offer speed advantages, future research should assess the usability of these paradigms in patient populations with visual impairments where atypical eye movements may pose challenges. For this, also investigating the test–retest repeatability of measurements should be established. If these methods can be proven to be reliable in clinical populations, they offer a potential tool for detecting and monitoring vision impairments such as glaucoma (Ogata et al., 2019) and understanding the daily challenges faced by individuals with dyslexia (Gori and Facoetti, 2015).

Additionally, we observed greater variability in crowding magnitude and orientation discrimination thresholds in the 6AFC and serial search paradigms. Exploring the factors contributing to this variability, such as individual differences in eye movement patterns or attentional strategies, could help refine these paradigms for clinical use. If indeed attentional processes differentially affect the crowding estimates (Scolari et al., 2007; Bacigalupo and Luck, 2015; Grillini et al., 2019) in the various paradigms, this demands a further scrutiny of their suitability for testing diverse populations. Investigating the specific attentional mechanisms involved in each paradigm and how they influence crowding measurements could provide valuable fundamental insights into crowding.

Furthermore, the utilization of calibration-free eye trackers could be a significant benefit for future studies, particularly in clinical settings and with diverse populations. Unlike traditional eye trackers that require calibration, calibration-free eye trackers offer potential advantages in terms of ease of use and time efficiency (Harezlak and Kasprowski, 2018). This technology could be particularly advantageous for patient populations with visual impairments, where traditional calibration routines may be challenging or impractical. Incorporating calibration-free eye trackers in future research could enhance the accessibility and applicability of eye movement-based crowding paradigms for clinical assessment.

While our study primarily focused on peripheral visual crowding, investigating foveal crowding is equally important. Previous studies indeed developed eye movement tests to assess foveal crowding (Tanke et al., 2021). Evaluating the reliability of such tests in detecting visual impairments such as amblyopia (Kalpadakis-Smith et al., 2022) could contribute to advancing the development of more efficient foveal crowding assessment techniques.

Finally, our intention to manipulate target-flanker similarity was constrained by the overly narrow range of spatial frequencies used. Future studies could again look into this aspect, and its influence on crowding. Furthermore, other stimulus parameters, such as spacing, eccentricity, and contrast, known to affect crowding magnitude could also be evaluated. For instance, investigating the impact of eccentricity variations on crowding across different paradigms could enhance our understanding of how visual processing differs in clinical populations. Assessing the influence of spacing and contrast variations on crowding can contribute to refining clinical assessments by optimizing stimulus parameters to better capture crowding effects. Systematically investigating these parameters across different paradigms not only expands our understanding of crowding phenomena but also provides concrete avenues for improving the accuracy and effectiveness of clinical assessments.

## 5 Conclusion

In conclusion, developing new, fast, and reliable paradigms that can be easily administered is crucial for the routine assessment of peripheral crowding. Paradigms that implement eye movements, particularly the serial search paradigm, and the 6AFC paradigm with eye movements, show promise in this regard, at least in young and healthy populations. Future research should explore the usability of these paradigms in patient groups with neurological and visual impairments.

## Data availability statement

The raw data supporting the conclusions of this article will be made available by the authors, without undue reservation.

## Ethics statement

The studies involving humans were approved by the Medical Ethics Review Board of the University Medical Center Groningen (METc UMCG). The studies were conducted in accordance with the local legislation and institutional requirements. The participants provided their written informed consent to participate in this study.

## Author contributions

DT: Conceptualization, Data curation, Formal analysis, Investigation, Methodology, Project administration, Software, Visualization, Writing – original draft. FC: Conceptualization, Funding acquisition, Methodology, Resources, Supervision, Writing – review & editing.
