# Supplementary material for: Rapid assessment of peripheral visual crowding
Source: Front Neurosci. 2024 Mar 25;18:1332701. doi: 10.3389/fnins.2024.1332701 (PMC11019380; doi:10.3389/fnins.2024.1332701)
Supplement: Supplementary file 1 [file Data_Sheet_1.docx]

*Table S1.* *Bayes Factors of main and interaction effects of Bayesian ANOVA on thresholds and crowding magnitude including the three participants with outliers*

| **Effects** | **Thresholds BF_included_** | **Crowding Magnitude BF_included_** |
| --- | --- | --- |
| Paradigm | 262716.66 | 2.97 |
|  |  |  |
| Condition | 5386.47 | 0.83 |
|  |  |  |
| Paradigm*Condition | 8.37 | 0.13 |
|  |  |  |

*Table S2. Average flanked (high similarity) and isolated thresholds in all paradigms including the three participants with outliers*

| **Paradigm** | **Condition** | **Mean** | **Posterior Odds** | **error%** |
| --- | --- | --- | --- | --- |
| 2AFC Eye Movement | Flanked | 9.40 | 1.61 | <0.01 |
|  | Isolated | 5.04 |  |  |
| 2AFC Manual | Flanked | 8.14 | 1.23 | <0.01 |
|  | Isolated | 6.12 |  |  |
| 6AFC Eye Movement | Flanked | 24.99 | 4.04 | <0.01 |
|  | Isolated | 17.82 |  |  |
| 6AFC Manual | Flanked | 23.14 | 25.54 | <0.01 |
|  | Isolated | 13.74 |  |  |
| Serial Search | Flanked | 33.31 | 21.81 | <0.01 |
|  | Isolated | 23.24 |  |  |

*Table S3. Post hoc comparisons of paradigms in terms of crowding magnitude including the three participants with outliers*

| **Comparisons** | | **Posterior Odds** | **error%** |
| --- | --- | --- | --- |
| 2AFC Eye Movement | 2AFC Manual | 0.08 | 0.03 |
|  | 6AFC Eye Movement | 0.72 | <0.01 |
|  | Serial Search | 0.56 | <0.01 |
| 2AFC Manual | 6AFC Manual | 19.43 | <0.01 |
|  | Serial Search | 3.35 | <0.01 |
| 6AFC Eye Movement | 6AFC Manual | 0.10 | 0.03 |
|  | Serial Search | 0.06 | 0.03 |
| 6AFC Manual | Serial Search | 0.08 | 0.03 |


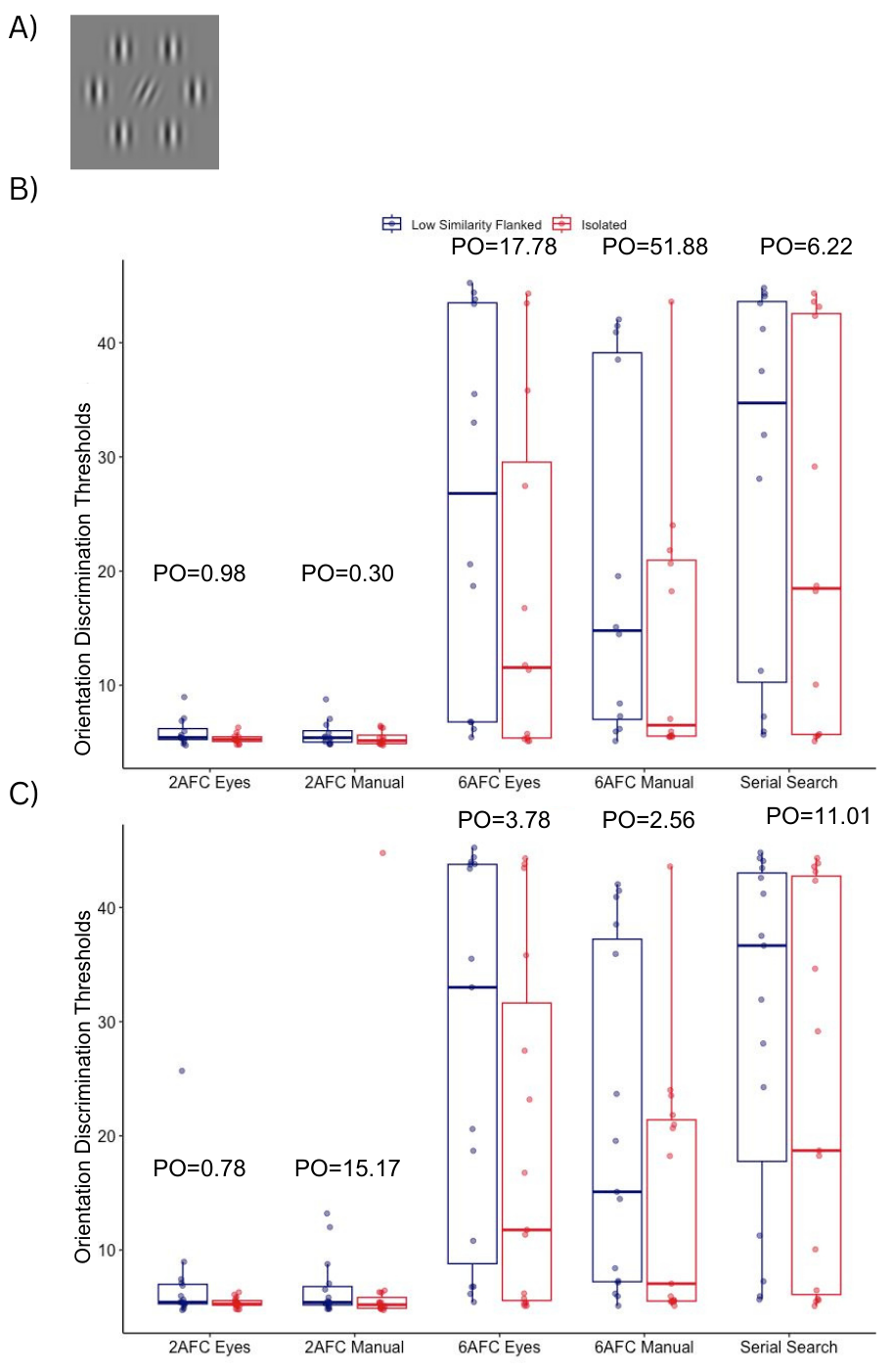


*Figure S1.* Stimulus and the data for the low target-flanker similarity condition. Figure S1A depicts the stimulus presented for the low target flanker similarity conditions. Flanker Gabor’s spatial frequency was set to 4 cycles per degree (cpd) to decrease their similarity to the target Gabor with 5 cpd spatial frequency. Figure S1B represents the orientation discrimination thresholds in five paradigms for low target-flanker similarity and isolated conditions for all 15 participants (including outliers). Posterior odds (PO) show the likelihood of the threshold of flanked condition thresholds being different from the isolated conditions. Figure S1C depicts the same values excluding the three participants with outliers.

*Table S4. Preference Questionnaire. Participants answered all of these questions for each paradigm after completion of all five paradigms.*

| 1.How would you assess the difficulty of the paradigm? | | | | |
| --- | --- | --- | --- | --- |
| 1 | 2 | 3 | 4 | 5 |
| Not difficult |  |  |  | Very difficult |

| 2.How tiring was the paradigm? | | | | |
| --- | --- | --- | --- | --- |
| 1 | 2 | 3 | 4 | 5 |
| Not tiring |  |  |  | Very tiring |

| 3.How demanding was the paradigm? | | | | |
| --- | --- | --- | --- | --- |
| 1 | 2 | 3 | 4 | 5 |
| Not demanding |  |  |  | Very demanding |

| 4.How much attention did the paradigm require? | | | | |
| --- | --- | --- | --- | --- |
| 1 | 2 | 3 | 4 | 5 |
| Not much |  |  |  | Too much |

| 5.How much effort did the paradigm require? | | | | |
| --- | --- | --- | --- | --- |
| 1 | 2 | 3 | 4 | 5 |
| Not much |  |  |  | Too much |

*Table S5. Paradigm comparison in terms of recalculated durations.*

| **Paradigm Comparison** | **PO_CI-1_** | **PO_CI-2_** | **Three Pt. Excluded** |
| --- | --- | --- | --- |
| 2AFC Eye Movement-Serial Search | 0.85 | 0.80 | 2.66 |
| 6AFC Eye Movement-Serial Search | 1.13 | 0.46 | 1.47 |
| 2AFC Manual-Serial Search | 28.98 | 17.97 | 14.39 |
| 6AFC Manual-Serial Search | 17.68 | 7.32 | 4.66 |
| 6AFC Manual-6AFC Eye Movement | 1.30 | 0.87 | 0.83 |
| 2AFC Manual-2AFC Eye Movement | 4.55 | 3.55 | 17.90 |

*The first two columns present posterior odds (PO) with confidence intervals: 19 (CI-1) and 20 (CI-2). The last column presents the re-calculated durations with an original CI of 18 but excludes the three participants who were excluded in the threshold and crowding magnitude analyses.*

*
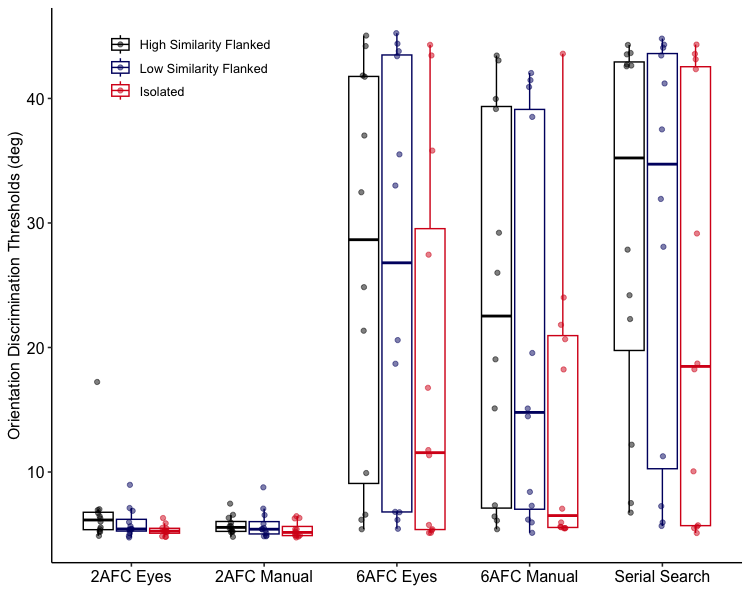
*

*Figure S2.* The recalculated orientation discrimination thresholds for flanked and isolated conditions using a cut-off point of 18 CI.

*Table S6. Bayes Factors of main and interaction effects of Bayesian ANOVA on thresholds and crowding magnitude for comparison of original and re-estimated values using CI value of 19 (CI-1) and 20 (CI-2).*

| **Effects** | **Threshold BF_CI-1_** | **Crowding Magnitude BF_CI-1_** | **Threshold BF_CI-2_** | **Crowding Magnitude BF_CI-2_** |
| --- | --- | --- | --- | --- |
| Threshold Calculation | 0.39 | 4.53 | 0.62 | 6.54 |
| Paradigm * Threshold Calculation | 0.85 | 0.78 | 1.72 | 2.68 |
| Condition * Threshold Calculation | 0.26 | 0.27 | 0.41 | 0.92 |
| Paradigm * Condition * Threshold Calculation | 0.23 | 0.001 | 0.12 | 3.81 |


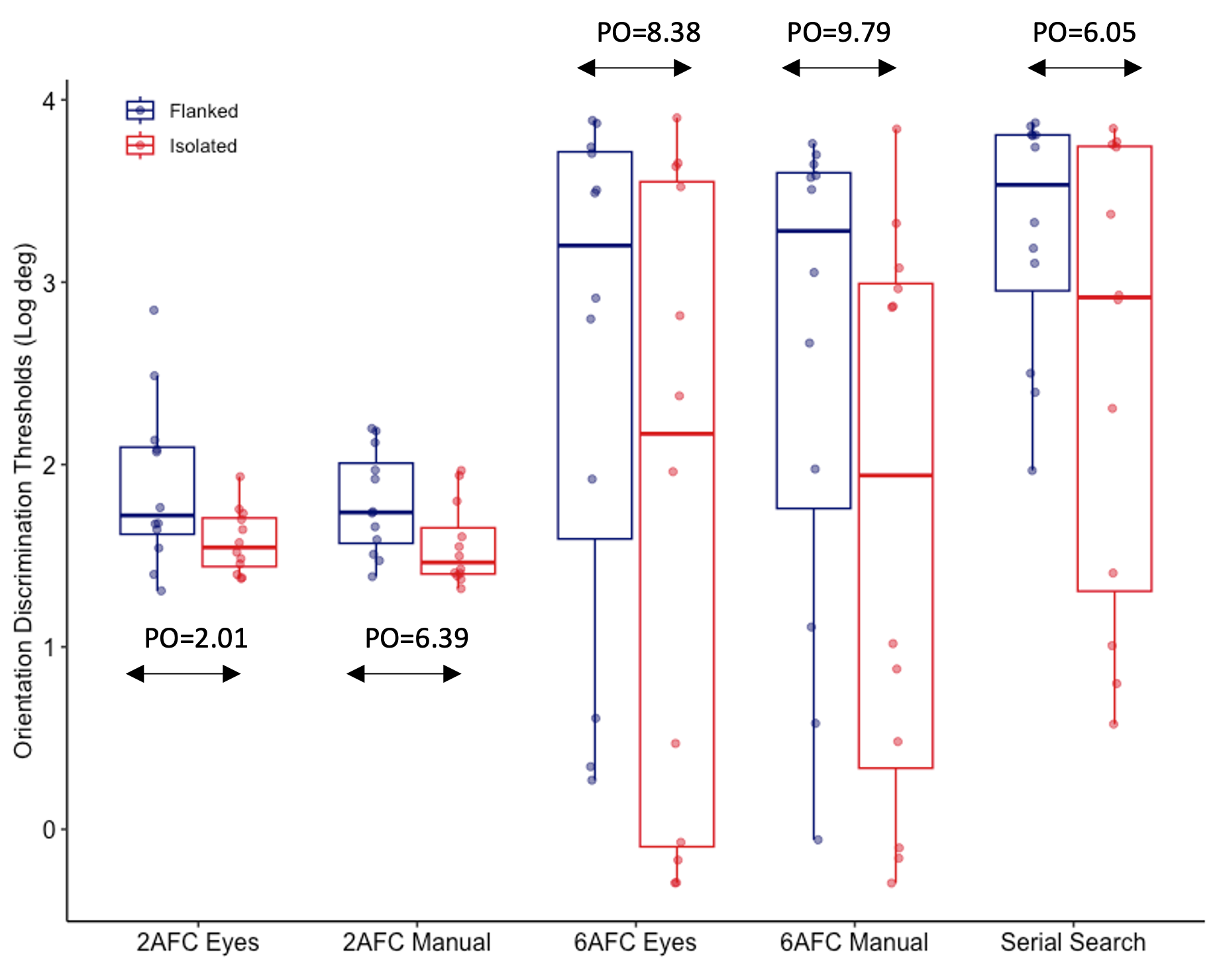


Figure S3. Log transformed isolated and flanked orientation discrimination thresholds obtained with all five paradigms. The figure shows flanked (high-similarity) orientation discrimination thresholds in blue and isolated orientation discrimination thresholds in red. “PO” represents the posterior odds of the flanked condition being different from the isolated condition for each paradigm.
